# Development and evaluation of case video-based debriefing on a simulation of high-risk neonatal care for nursing students in South Korea: a mixed-methods study

**DOI:** 10.1186/s12912-023-01507-2

**Published:** 2023-09-27

**Authors:** Hyun Young Koo, Bo Ryeong Lee, Hyeran An

**Affiliations:** 1 College of Nursing, Research Institute of Nursing Science, Daegu Catholic University, 33 Duryugongwon-Ro, 17 Gil, Nam-Gu, Daegu, 42472 Korea; 2 College of Nursing, Daegu Catholic University, 33 Duryugongwon-Ro, 17 Gil, Nam-Gu, Daegu, 42472 Korea

**Keywords:** Simulation training, Intensive care unit, Neonatal, Infant, Newborn, Students, Nursing

## Abstract

**Background:**

The debriefing process after health care simulations should provide a psychologically safe learning environment for nursing students. Case video-based debriefing on a simulation of high-risk neonatal care can help nursing students feel psychologically safe and make learning more effective. In this study, we developed case video-based debriefing materials for a simulation of high-risk neonatal care for nursing students in South Korea and evaluated their effects.

**Methods:**

This mixed-methods study, consisting of a survey and an in-depth interview, was conducted between August and December 2022. The participants were 27 nursing students for the development of the case video-based debriefing and 51 nursing students for the evaluation of its effects (25 in the experimental group and 26 in the control group) at a university in South Korea. A case video-based debriefing on a simulation of high-risk neonatal care was developed, and the experimental group took part in case video-based debriefing. The participants’ self-efficacy, critical thinking, state anxiety, and satisfaction with practice were examined. The experimental group’s learning experiences were explored. Quantitative data were analyzed using the chi-square test, the unpaired t-test, and repeated-measures analysis of variance. Qualitative content analysis was conducted.

**Results:**

In the experimental group, critical thinking and satisfaction with practice increased to a greater extent than in the control group. However, the changes in self-efficacy and state anxiety were not significantly different between the experimental and control groups. Four categories were extracted from nursing students who participated in the case video-based debriefing: “learning facilitated by the simulation,” “expanded learning,” “safe learning environment,” and “efficient utilization of case videos.”

**Conclusions:**

Case video-based debriefing on a simulation of high-risk neonatal care effectively enhanced nursing students’ critical thinking and satisfaction with practice, and it will be utilized to improve nursing students’ competency in high-risk neonatal care.

## Background

For nursing students to become competent in high-risk neonatal care in the clinical field after graduation, they must gain experience discovering and solving neonatal care problems by applying theoretical nursing knowledge to an authentic clinical context [1]. Due to recent changes in the medical field, such as measures implemented in response to emerging infectious diseases, and an increase in the number of premature births [2], the quantity and difficulty of nursing work have increased, requiring nurses to have a broader range of competencies. However, opportunities for nursing students to practice nursing competencies have decreased due to practice restrictions aiming to protect vulnerable patients [3, 4]. Various nursing education methods are being put into place to overcome these difficulties [5], and it is necessary to develop and implement effective educational methods considering the reality that opportunities to practice nursing for vulnerable newborns have been restricted.

Simulations are an effective educational method that helps nursing students deal with realistic clinical cases and perform nursing activities in a safe environment [6]. Simulations allow nursing students to repeat nursing care activities that are difficult to experience in the clinical field through high-fidelity simulators and/or standardized patients [7]. Previous studies have found that nursing students who took part in simulations were able to develop nursing skills in safe and controlled environments [8, 9] and exhibited enhanced learning outcomes [10].

Health care simulations consist of three stages: pre-briefing, which is a preparatory process; simulation running, which proceeds according to the learning goals; and debriefing, in which learners reflect on their performance and receive feedback [11]. Debriefing is a key step that can increase learning performance, in which learners reflect on their performance in the simulation and gain confidence that they can appropriately respond without making the same mistakes in repeated situations [12]. Various debriefing methods exist, such as video debriefing, verbal debriefing, and debriefing with a model of clinical judgment [13]. Video debriefing helps students improve their self-efficacy by allowing them to reflect on their own behavior based on a direct video recording of what they have performed in a simulated situation, without relying on recall [14]. Video learning materials are easy to remember, promote thinking, and help focus; furthermore, they enable the simultaneous integration of individual learning that takes into account individual differences with cooperative learning involving other learners, thereby providing a rich learning environment [15]. Video debriefing improves learners’ knowledge, performance confidence, and clinical critical thinking; furthermore, compared to verbal debriefing, it is also more effective in enhancing satisfaction with learning and clinical performance ability [16].

In previous research, video debriefing seemed to induce learners to act more appropriately based on direct observations of their actions [17], but learners reported that they were nervous and uncomfortable, as they felt like someone was constantly watching them while their practice at the simulation center was recorded [18]. A systematic review on the effectiveness of the video debriefing method in simulation practice training [19] found no significant effect using video debriefings, except for a simulation of CPR, among 11 studies using video for debriefings [20]. When participating in simulations, learners felt nervous about practicing in front of instructors and other students, making mistakes, and being recorded [18]. In addition, the learners felt embarrassment and stress while watching the video recordings during the debriefing with the instructor and other students [21]. Therefore, instructors should strive to increase the effectiveness of simulation learning by maintaining the advantages of video learning materials while taking steps to reduce learners’ anxiety. Previous studies [14, 17, 22, 23] on video debriefing used video recordings of learners' processes performed in simulations for debriefing. In this study, various high-risk neonatal care simulation situations were produced in advance for different topics and used in the debriefing sessions to create a safe learning environment by reducing learners’ tension during the simulation process and their embarrassment and stress during the debriefing process. A psychologically secure environment for simulations reduces learners’ anxiety and increases their self-esteem; therefore, learners feel a higher degree of satisfaction with the simulation practice [24].

Therefore, the first aim of this study was to identify the mistakes that learners often make in a simulation of high-risk neonatal care and to utilize that information to produce video clips for debriefing. The case video-based debriefing on a simulation of high-risk neonatal care was intended to help nursing students feel psychologically safe and make their learning more effective. Next, this study examined the effects of case video-based debriefing on self-efficacy, critical thinking, state anxiety, and satisfaction with practice. In addition, this study explored students’ learning experiences during case video-based debriefing.

The research hypotheses were as follows: first, self-efficacy would increase to a greater extent in the experimental group than in the control group; second, critical thinking would increase to a greater extent in the experimental group than in the control group; third, state anxiety would show a greater decrease in the experimental group than in the control group; and fourth, satisfaction with practice would increase to a greater extent in the experimental group than in the control group.

## Methods

### Study design

**Fig. 1:**
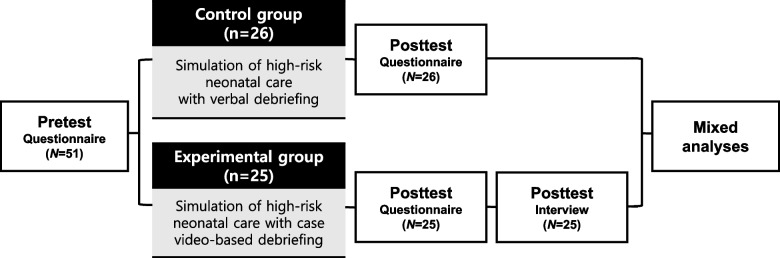
Research design

This was a mixed-methods study consisting of a survey for quantitative data and an in-depth interview for qualitative data. To evaluate the effects of case video-based debriefing, a quasi-experimental study was conducted with a nonequivalent control group pretest–posttest design at one university in South Korea (Fig. 1). This study adhered to the Transparent Reporting of Evaluations with Nonrandomized Designs (TREND) reporting guidelines [25].

### Setting and sample

Students at a nursing college in South Korea were recruited through convenience sampling. Nursing students who voluntarily consented to take part in the study in writing were included from August to December 2022. To develop case video-based debriefing, the inclusion criterion was having experienced a simulation of high-risk neonatal care, and the exclusion criteria were refusing to participate in the study or not having experienced a simulation of high-risk neonatal care. To evaluate the case video-based debriefing, the inclusion criterion was participating in a 45-h, one-credit neonatal intensive care unit (NICU) practicum, and the exclusion criteria were refusing to participate in the study or not completing a NICU practicum.

The G*Power 3.1.9.7 program with repeated-measures analysis of variance (ANOVA) and within-between interactions was used to calculate the number of participants. An effect size of 0.25, which was based on previous research [7], a significance level of 0.05, a power of 80%, a correlation coefficient of 0.30, and a number of measurements of 2 were entered, and the resulting sample size was 23 participants for each group [26]. Taking a 10% dropout rate into account, 52 students (26 in each group) were recruited. Due to a vacancy caused by a private scheduling conflict, the study finally included 25 participants in the experimental group and 26 participants in the control group, with no additional dropouts.

The nursing students were divided into eight teams, consisting of 6–7 members, and one team per week participated in the NICU practicum. To prevent the experimental effect from spreading, students who practiced in the NICU during the first half of the practicum were put into the control group, while students who practiced in the NICU during the second half were allocated to the experimental group.

### Development and evaluation of case video-based debriefing on a simulation of high-risk neonatal care

Case video-based debriefing on a simulation of high-risk neonatal care was developed using the analysis, design, development, implementation, and evaluation (ADDIE) model [27].

#### Analysis

A questionnaire survey and focus group interviews were conducted among 27 nursing students who had previously participated in simulations of high-risk neonatal care. In the open-ended questionnaire survey, the participants were asked to describe what they did wrong (situations where they made mistakes), difficult situations, most memorable situations, most important points, and most memorable debriefing experiences in simulations of high-risk neonatal care. Five focus groups consisting of 5–6 participants each were formed, and one interview was conducted per group. The focus group interviews were conducted in a small conference room at convenient times for the participants. They were asked about the same topics covered in the questionnaire. With prior consent, the interviews were recorded and transcribed verbatim immediately after interview completion.

#### Design

Based on the results of the open-ended questionnaire survey and the contents of the focus group interviews, a case video scenario for debriefing was created. The scenario was developed based on cases in which nursing students made many mistakes, cases that they found difficult, and cases that they considered important. The scenario was completed through a validity test conducted by one nursing professor with experience in operating simulations of high-risk neonatal care, one nurse with more than 5 years of NICU work experience, and two nursing students with experience in simulations of high-risk neonatal care (Table 1).

**Table 1:** The contents of the debriefing scenario on a simulation of high-risk neonatal care

**No**	**Scenario topics**	**Contents**
1	Initial assessment of neonatal respiration	In the initial assessment, respiratory function-related diagnostic test results were not reviewed and lung sounds were not auscultated
2	Interpretation of lung sounds	The student was confused by misinterpreting normal lung sounds as abnormal lung sounds during the initial assessment and after endotracheal suction
3	Interpreting patient monitor alarms	When a patient monitor alarm sounded for airway obstruction due to apnea or sputum, only SpO_2_ levels were checked and only tactile stimuli were provided
4	Airway positioning	A low tidal volume alarm sounded because a high-risk neonate was placed in hyper-extension position
5	Aseptic procedure during endotracheal suction	Hands wearing sterile gloves, a suction catheter, and the ventilator circuit were contaminated during endotracheal suction and hands were not washed after suction
6	Ventilation during endotracheal suction	Endotracheal suction was continued when SpO_2_ dropped below 90% and the heart rate dropped, so SpO_2_ and the heart rate did not recover
7	Role of the leader	The intervention was incorrect or delayed because the charge nurse focused only on nursing records, without giving proper instructions or help to other members

*SpO*_*2*_ Saturation of percutaneous oxygen

#### Development

Based on the completed scenario, a video of about 5 min for each topic was produced. The produced case video was completed after validity testing conducted by one nursing professor with experience in operating simulations of high-risk neonatal care, one nurse with more than 5 years of NICU work experience, and two nursing students with experience in participating in simulations of high-risk neonatal care, who also verified the validity of the scenario.

#### Implementation

From September to October 2022, verbal debriefing was performed for the control group, in which the instructor provided verbal feedback in the debriefing stage of a simulation of high-risk neonatal care, and from November to December 2022, debriefing was performed using the developed case videos for the experimental group. Debriefing was conducted by showing a case video where the experimental group made mistakes or incorrectly implemented the simulation, and an average of four case videos were used for each practice group. According to the clinical practice operation regulations, the same practice location, practice period, and practice guidance were applied to both the control group and the experimental group. The participants were not told which group they were assigned to.

#### Evaluation

In both the control and experimental groups, general characteristics, self-efficacy, critical thinking, state anxiety, and satisfaction with practice were investigated 1–2 days before the start of NICU practice and on the last day of NICU practice after the practice was completed. The time required for the survey was approximately 20 min. The researchers did not directly conduct the pre- and post-surveys and were blinded, since questionnaires were distributed by the head of the practice group and participants were requested to provide self-reported answers. Each questionnaire had a unique number that the study participants were asked to remember when filling out the pre-survey questionnaire, and they were asked to fill out the post-survey questionnaire with the same unique number. If there were missing answers in the questionnaire, the head of the practice group asked study participants who remembered their unique numbers to answer the corresponding questions again; thus, there were no questionnaires with missing answers.

The experimental group completed the post-survey questionnaire on the last day of NICU practice and participated in focus group interviews. One researcher who did not participate in conducting the simulation formed focus groups, each of which consisted of 6–7 members of the practice team, and conducted interviews in a small conference room. The participants were asked to discuss the positive and negative points of their debriefing experience using case videos for the simulation of high-risk neonatal care and differences from previous experiences of verbal debriefing. With prior consent, the interviews were recorded and transcribed verbatim immediately after interview completion.

### Instruments

All instruments were designed as self-reported surveys and were utilized only after the original authors’ agreement.

#### Self-efficacy

Self-efficacy was measured using the general self-efficacy scale for adults, which was developed by [28]. On a 6-point Likert scale, 10 items were measured (1: strongly disagree, 6: strongly agree). A higher score indicated better self-efficacy. Cronbach’s α was 0.87 in the study of Song [28], 0.90 in the pretest of this study, and 0.93 in the posttest of this study.

#### Critical thinking

Critical thinking was measured using the critical thinking disposition scale, which was developed by Yoon [29]. On a 5-point Likert scale, 27 items were measured (1: strongly disagree, 5: strongly agree). A higher score indicated better critical thinking when negative items were reverse-coded and totaled. Cronbach’s α was 0.84 in the study of Yoon [29], 0.85 in the pretest of this study, and 0.88 in the posttest of this study.

#### State anxiety

State anxiety was measured using the state anxiety scale of the state-trait anxiety inventory, which was developed by Spielberger [30] and translated into Korean by Kim & Shin [31]. On a 4-point Likert scale, 20 items were measured (1: strongly disagree, 4: strongly agree). A higher score corresponded to higher anxiety when negative items were reverse-coded and totaled. Cronbach’s α was 0.92 in the study of Spielberger [30] and 0.93 in both the pretest and posttest of this study.

#### Satisfaction with practice

Satisfaction with practice was measured using the scale developed by Yoo [32], which was revised by Chang and Park [33]. On a 5-point Likert scale, 17 items were measured (1: strongly disagree, 5: strongly agree). A higher score revealed better satisfaction with practice. Cronbach’s α was 0.86 in the study of Chang and Park [33], 0.95 in the pretest of this study, and 0.93 in the posttest of this study.

### Data analysis

The quantitative data were analyzed using SPSS version 25.0 (IBM Corp., Armonk, NY, USA). The chi-square test and the unpaired t-test were used to assess baseline homogeneity between the experimental and control groups. Repeated-measures ANOVA was used to examine changes in outcome variables based on the intervention.

The qualitative data were analyzed using the inductive approach of content analysis suggested by Elo and Kyngas [34]. During the preparation phase, all data were grasped by reviewing each interview topic several times. During the categorization phase, sentences that reflected the experiences of the participants were chosen as the analytic unit via open coding and read numerous times to extract meaningful assertions. Similar materials were grouped together to establish subcategories, which were further abstracted to form categories. During the reporting phase, the categories were presented.

## Results

### Homogeneity testing of participants’ general characteristics

There were no significant differences in general characteristics between the experimental and control groups, including age, gender, academic performance, health status, and satisfaction with university life. There were also no significant differences between the experimental and control groups in self-efficacy, critical thinking, state anxiety, or satisfaction with practice (Table 2).

**Table 2:** Homogeneity testing of the general characteristics and outcome variables of the two groups

Variables or categories	Total (*N* = 51)	Exp. (*n* = 25)	Cont. (*n* = 26)	X^2^/t	*p*
	n (%) or Mean ± SD	n (%) or Mean ± SD	n (%) or Mean ± SD		
Age (year)	21.76 ± 1.21	21.84 ± 1.11	21.69 ± 1.32	0.432	.667
Gender					
Male	11 (21.6)	5 (20.0)	6 (23.1)	0.071	.789
Female	40 (78.4)	20 (80.0)	20 (76.9)		
Academic performance (percentile)					
≤ 30	13 (25.5)	6 (24.0)	7 (26.9)	0.057	.811
> 30	38 (74.5)	19 (76.0)	19 (73.1)		
Health status					
Healthy	36 (70.6)	18 (72.0)	18 (69.2)	0.047	.828
Unhealthy	15 (29.4)	7 (28.0)	8 (30.8)		
Satisfaction with university life					
Satisfied	38 (74.5)	19 (76.0)	19 (73.1)	0.057	.811
Unsatisfied	13 (25.5)	6 (24.0)	7 (26.9)		
Self-efficacy	44.75 ± 5.93	45.76 ± 5.21	43.77 ± 6.50	1.203	.235
Critical thinking	100.16 ± 9.01	99.72 ± 10.48	100.58 ± 7.52	0.337	.738
State anxiety	44.67 ± 10.93	42.16 ± 11.68	47.08 ± 9.77	1.633	.109
Satisfaction with practice	71.25 ± 9.44	72.52 ± 10.43	70.04 ± 8.40	0.938	.353

*Cont.* Control group, *Exp.* Experimental group

### The effects of case video-based debriefing on a simulation of high-risk neonatal care

There was a significant difference in self-efficacy by group (F = 4.28, *p* = 0.044). However, there was no significant difference between the groups according to time (F = 2.15, *p* = 0.149), nor was there a difference according to time alone (F = 1.20, *p* = 0.279) (Table 3). In other words, the experimental and control groups’ patterns of change following the intervention did not differ significantly, disproving hypothesis 1.

**Table 3:** Self-efficacy, critical thinking, state anxiety, and satisfaction with practice between the two groups

Variables or groups	Pretest	Posttest	Source	t or F	*p*
	Mean ± SD	Mean ± SD			
Self-efficacy					
Exp	45.76 ± 5.21	47.88 ± 6.78	G	4.277	.044
Cont	43.77 ± 6.50	43.46 ± 6.46	T	1.197	.279
			G*T	2.147	.149
Critical thinking					
Exp	99.72 ± 10.48	106.60 ± 10.90	G	0.587	.447
Cont	100.58 ± 7.52	102.19 ± 8.03	T	12.455	.001
			G*T	4.783	.034
State anxiety					
Exp	42.16 ± 11.68	39.28 ± 9.89	G	2.967	.091
Cont	47.08 ± 9.77	44.00 ± 11.26	T	7.891	.007
			G*T	0.009	.926
Satisfaction with practice					
Exp	72.52 ± 10.43	79.88 ± 5.97	G	4.838	.033
Cont	70.04 ± 8.40	73.12 ± 7.84	T	27.281	< .001
			G*T	4.594	.037

*Cont.* Control group, *Exp.* Experimental group, *G* group, *T* time, G*T. Differences between groups over time

There was no significant difference in critical thinking by group (F = 0.59, *p* = 0.447). However, significant differences were found according to time (F = 12.46, *p* = 0.001) and between the groups according to time (F = 4.78, *p* = 0.034) (Table 3). Thus, our second hypothesis was supported by the finding that critical thinking improved more in the experimental group than in the control group.

State anxiety showed a significant difference by time (F = 7.89, *p* = 0.007), but there was no significant difference between the groups according to time (F = 0.01, *p* = 0.926), nor was there a difference by group alone (F = 2.97, *p* = 0.091) (Table 3), In other words, the patterns of change after the intervention did not significantly differ between the experimental group and the control group, refuting hypothesis 3.

Satisfaction with practice showed a significant difference by group (F = 4.84, *p* = 0.033), as well as by time (F = 27.28, *p* < 0.001) and according to both group and time (F = 4.59, *p* = 0.037) (Table 3). Our fourth hypothesis was supported by the finding that satisfaction with practice improved more in the experimental group than in the control group.

### The experiences of case video-based debriefing on a simulation of high-risk neonatal care

Four categories and 10 subcategories were extracted through the data analysis. The subcategories “the material became easier to understand,” “etched in memory,” and “immersion in learning” were classified as belonging to the first category, “learning facilitated by the simulation.” The subcategories “identified unrecognized mistakes” and “acquired a new problem-solving method” comprised the second category, “expanded learning.” The subcategories “free from the stress of evaluation,” “free from social pressure related to the evaluation,” and “accepted the evaluations objectively” constituted the third category, “safe learning environment.” The subcategories “limitations of case videos” and “development of videos for various cases” were components of the fourth category, “efficient utilization of case videos” (Table 4).

**Table 4:** Experiences of case video-based debriefing on a simulation of high-risk neonatal care: categories and subcategories

Categories	Subcategories
Learning facilitated by the simulation	The material became easier to understand
	Etched in memory
	Immersion in learning
Expanded learning	Identified unrecognized mistakes
	Acquired a new problem-solving method
Safe learning environment	Free from the stress of evaluation
	Free from social pressure related to the evaluation
	Accepted the evaluations objectively
Efficient utilization of case videos	Limitations of case videos
	Development of videos for various cases

#### Category 1. Learning facilitated by the simulation

##### Subcategory 1. The material became easier to understand

Participants said that they did not understand their actions in the simulation based on the instructor’s explanation alone. Instead, they were able to understand their actions more easily and accurately by watching case videos.

“*There were some parts that I couldn’t understand just from the professor’s explanation, but after watching the video, I understood them right away.*” (Participant 4)

##### Subcategory 2. Etched in memory

Participants said that after finishing the simulation in a tense state, they did not remember exactly what they and the members of the group did during the simulation. They said that by watching the case video in the debriefing stage, they clearly remembered what they did in the simulation. They were able to have detailed discussions and receive feedback based on accurate memories of the simulations they experienced and they could remember the contents clearly.

“*I was so nervous during the simulation that I couldn’t remember a thing after it was over, but after watching the case video, I remembered it again, so it was nice. Also, while watching the case video, the professor pointed out my mistake, and I was able to talk with the professor and team members about how to act in the future, so it was more memorable.*” (Participant 11)

##### Subcategory 3. Immersion in learning

Participants were more immersed in learning because the environment and situation in the case video matched the environment and situation when they performed the simulation.

“*The environment in the video and the environment in the simulation were the same, and the mistakes shown in the video were the same as the mistakes we made. That’s why I was able to empathize tremendously and was able to immerse myself during the debriefing.*” (Participant 19)

#### Category 2. Expanded learning

##### Subcategory 1. Identified unrecognized mistakes

Participants were able to identify various mistakes they could have made through case videos. They had an opportunity to learn to prevent future mistakes.

“*I could make a mistake like that at any time. While watching the video, I thought, ‘I can make mistakes like this’ and ‘I can make mistakes like that.’ In the simulation, I could see what I should be careful about in the future.*” (Participant 25)

##### Subcategory 2. Acquired a new problem-solving method

Participants confirmed in detail where they made a mistake through case videos produced for various mistakes that could occur in the simulation. They also realized from the case videos that there was a solution other than how they solved the problem.

“*I learned that doing that is a mistake, and I also learned that problems can be solved differently.*” (Participant 2)

#### Category 3. Safe learning environment

##### Subcategory 1. Free from the stress of evaluation

Participants were nervous and embarrassed when they were evaluated by instructors and team members on what they did well or wrong while watching the video of their simulation during debriefing in other subjects. However, in this simulation, they were able to immerse themselves in learning comfortably as debriefing proceeded while watching a case video of others who took their roles.

“*I was so embarrassed when I watched the video with other people, but I was relieved because I wasn’t appearing in this video.*” (Participant 3)

##### Subcategories 2. Free from social pressure related to the evaluation

During the debriefing, participants said that if they pointed out another peer’s mistakes, they did not express their opinions actively, fearing that the pointed-out peer would be upset. However, in this debriefing using case videos in which they and their peers did not appear, they were able to express their opinions freely without the need to worry about their peers’ feelings.

“*It was hard to point out my friend’s mistake while watching their video. If I point out my friend’s mistake, they might feel bad, so I didn’t say anything. But this time, I was able to talk comfortably about their mistakes with them.*“ (Participant 1)

##### Subcategory 3. Accepted the evaluations objectively

Participants were able to focus on the mistake itself, not the person who made the mistake, in the debriefing through case videos featuring other people replacing their roles. As a result, they were able to accept feedback comfortably without emotional exhaustion.

“*The focus was on what went wrong, not who among us was at fault, so we were comfortable taking feedback.*” (Participant 21)

#### Category 4. Efficient utilization of case videos

##### Subcategory 1. Limitations of case videos

Participants said that it was difficult to concentrate on case videos that contained situations that did not exactly match the situations in which they made mistakes, so they did not clearly remember them. In addition, they were disappointed that the debriefing included discussion and feedback about their mistakes that were not included in the case videos.

“*It wasn’t a video that could point out exactly what we did wrong, so it wasn’t memorable*.” (Participant 25)

“*It was a pity that we didn’t have video materials for all the actions we did wrong.*” (Participant 20)

##### Subcategory 2. Development of videos for various cases

Participants were able to concentrate more on the debriefing through the case videos, and hoped that more diverse case videos would be developed.

“*I lost my immersion when there were discussions or feedback without videos. It would be nice if there were more examples of videos.*” (Participant 23)

## Discussion

In this study, we created case videos for debriefing based on the results of an open-ended questionnaire survey and focus group interviews among nursing students. The topics of the case videos were newborn assessment methods, interpretation of results, and basic assessment data, including aseptic techniques. Practical educational materials were created after analyzing the needs of nursing students, who were the intended audience of the materials. A study on educational needs for practicing neonatal intensive care among nursing students [35] also demonstrated a high level of educational needs for newborn assessments, reflecting the importance of accurately assessing high-risk newborns in the NICU. In other words, when conducting a simulation of high-risk neonatal care, nursing students should be instructed to be fully aware of the methods for accurately assessing and interpreting the condition of a newborn baby.

The experimental group that participated in the case video-based debriefing developed in this study showed a greater improvement in critical thinking than was observed in the control group. In the focus group interviews with the experimental group, participants reported that they were able to understand nursing activities more easily and accurately by watching case videos and that they were able to engage in detailed discussions and feedback because they remembered their own behavior. A previous study [22] also reported that videos provided nursing students with an opportunity to review what they had done and helped them reflect on their actions. Furthermore, it was also reported that videos improved nursing students’ ability to access materials for learning clinical skills [23]. In our study, case video-based debriefing enabled nursing students to identify mistakes they had not previously thought of and recognize new solutions, and the students thought that they make fewer mistakes in the future. In other words, using case videos in the debriefing stage made learning clearer and more comprehensive, resulting in improved critical thinking among the nursing students.

Satisfaction with practice also showed a greater increase in the experimental group than in the control group. Based on the focus group interviews with the experimental group, case video-based debriefing created a safe learning atmosphere. Conducting the debriefing while watching one’s own and colleagues’ simulation made students nervous about being evaluated by their instructors and peers and put them in the stressful social situation of having to evaluate their peers. This is consistent with a previous report [18] where the study participants felt nervous and stressed about recording a simulation. However, debriefing using case videos filmed as substitutes for analyzing their own and their team members’ actions helped them feel psychologically safe and introspect objectively without experiencing tension. This objectivity reduced unnecessary emotional stress and increased learners’ satisfaction with practice by allowing them to focus on the wrong action itself rather than the person who did something wrong.

However, self-efficacy and state anxiety were not found to differ over time between the experimental group and the control group. The self-efficacy of the control group did not change in the pre- and post-survey, and the self-efficacy of the experimental group increased in the post-survey compared to the pre-survey, but not to a significant extent. Self-efficacy refers to the belief that one can solve a problem by taking appropriate action in a specific situation [28]. Considering the results of previous studies, according to which interventions for self-efficacy last for at least 6 to 8 weeks [36], educational programs involving multiple sessions should be provided, and studies on long-term effects are needed.

In addition, there was no significant difference in state anxiety over time between the two groups, since state anxiety decreased from the pre-survey to the post-survey in both the experimental group and the control group. Nursing students who are about to take a practicum and simulation often experience anxiety, worrying about whether they will be able to do their job well without making mistakes. As state anxiety measures the present anxiety felt in a specific situation [30], it tends to be high just before a given task and decreases after the task is finished. Accordingly, it is reasonable that state anxiety decreased after the end of NICU practice in both groups. Therefore, it would be more appropriate to closely explore participants’ emotional states while participating in case video-based debriefing, and to do so, we also collected qualitative data in this study. The qualitative analysis confirmed that the experimental group comfortably immersed themselves in learning, free from stress about evaluation. In other words, debriefing using case videos provided nursing students with a safe learning atmosphere and facilitated learning.

In summary, this study developed case video-based debriefing materials for a simulation of high-risk neonatal care, and the experimental group took part in case video-based debriefing. Case video-based debriefing on a high-risk neonatal care simulation effectively improved nursing students’ critical thinking and satisfaction with practice. The use of case video-based debriefing will help nursing students become more proficient at providing high-risk neonatal care.

Nonetheless, there are several limitations to this study. First, nursing students from a single nursing college were recruited through convenience sampling, so its findings should be interpreted with caution. Second, in order to prevent the spread of the experimental effect, the experimental group and the control group were assigned according to the practice period; thus, random assignment was not performed. There were no significant differences in homogeneity testing of the general characteristics and dependent variables of the experimental group and the control group before the intervention, but a randomized controlled study is needed in the future. Third, despite our efforts to produce videos that matched the simulation situation, the video content had certain limitations. In particular, participants reported that it was difficult to concentrate on videos that did not exactly match their behavior. In the future, it will be necessary to develop videos of a broader range of cases and improve the video data. Fourth, only one posttest in this study was administered following the intervention, and no follow-up tests were administered to evaluate the long-term effect of case video-based debriefing. In the future, follow-up studies should be carried out to confirm the intervention’s long-term effects.

## Conclusions

This study developed case video-based debriefing materials for a simulation of high-risk neonatal care for nursing students and assessed its effects. The results revealed that case video-based debriefing effectively improved students’ critical thinking and satisfaction with practice. The application of case video-based debriefing to a simulation improved the learning effect for nursing students by providing a psychologically safe learning environment. Case video-based debriefing will be utilized to improve nursing students’ competency in high-risk neonatal care.

## Data Availability

The datasets are available from the corresponding author on reasonable request.

## Abbreviations

NICU: Neonatal intensive care unit
ANOVA: Analysis of variance
ADDIE: Analysis, design, development, implementation, and evaluation model
SpO_2_: Saturation of percutaneous oxygen
Cont.: Control group
Exp.: Experimental group
